# Sol–Gel Synthesis, Physico-Chemical and Biological Characterization of Cerium Oxide/Polyallylamine Nanoparticles

**DOI:** 10.3390/polym12071444

**Published:** 2020-06-28

**Authors:** Motaharesadat Hosseini, Issa Amjadi, Mohammad Mohajeri, Masoud Mozafari

**Affiliations:** 1 Department of Biomedical Engineering, Amirkabir University of Technology, Tehran 144961-4535, Iran; hosseini181@gmail.com; 2 Department of Biomedical Engineering, Wayne State University, Detroit, MI 84202, USA; 3 Department of Medical Biotechnology, Mashhad University of Medical Sciences, Mashhad 91336, Iran; mohammad.mohajeri@gmail.com; 4 Department of Tissue Engineering and Regenerative Medicine, Faculty of Advanced Technologies in Medicine, Iran University of Medical Sciences, Tehran 144961-4535, Iran; mozafari.masoud@gmail.com

**Keywords:** nanoceria, cancer cells, hemolytic activity, nanomedicine

## Abstract

Cerium oxide nanoparticles (CeO_2_-NPs) have great applications in different industries, including nanomedicine. However, some studies report CeO_2_-NPs-related toxicity issues that limit their usage and efficiency. In this study, the sol–gel method was applied to the synthesis of CeO_2_-NPs using poly(allylamine) (PAA) as a capping and/or stabilizing agent. The different molecular weights of PAA (15,000, 17,000, and 65,000 g/mol) were used to investigate the physico-chemical and biological properties of the NPs. In order to understand their performance as an anticancer agent, three cell lines (MCF7, HeLa, and erythrocyte) were analyzed by MTT assay and RBC hemolysis assay. The results showed that the CeO_2_-NPs had anticancer effects on the viability of MCF7 cells with half-maximal inhibitory concentration (IC50) values of 17.44 ± 7.32, 6.17 ± 1.68, and 0.12 ± 0.03 μg/mL for PAA15000, PAA17000, PAA65000, respectively. As for HeLa cells, IC50 values reduced considerably to 8.09 ± 1.55, 2.11 ± 0.33, and 0.20 ± 0.01 μg/mL, in order. A decrease in the viability of cancer cells was associated with the 50% hemolytic concentration (HC50) of 0.022 ± 0.001 mg/mL for PAA15000, 3.74 ± 0.58 mg/mL for PAA17000, and 7.35 ± 1.32 mg/mL for PAA65000. Ultraviolet-Visible (UV-vis) spectroscopy indicated that an increase in the PAA molecular weight led to a blue shift in the bandgap and high amounts of Ce^3+^ on the surface of the nanoceria. Thus, PAA65000 could be considered as a biocompatible nanoengineered biomaterial for potential applications in cancer nanomedicine.

## 1. Introduction

Nanotechnology, after nearly three decades, has opened up plenty of opportunities for the evolution of multifarious advanced materials in numerous products with prospective biomedical applications [1,2,3]. Metal centers are considered a key element of solids with extensive 2D or 3D structures and also biological macromolecules that render excellent properties and functions [4]. In this regard, cerium oxide nanoparticles (CeO_2_-NPs) were of great interest in medicine [5,6,7,8]. Cerium belongs to the lanthanide series that exists in abundance on the earth’s surface, at up to 0.0046%, and has the highest water solubility among the rare earth elements. It is the most reactive rare earth element after europium. Cerium is a potent oxidizing agent that can be rapidly oxidized when exposed to atmospheric oxygen [9,10]. Cerium is present in both the tetravalent (Ce^4+^) and trivalent (Ce^3+^) states. The following synonyms are frequently used for CeO_2_: Cerium dioxide, cerium oxide, cerium (IV) oxide, ceric oxide, and ceria [9]. CeO_2_ is applied to a miscellany of purposes, such as semiconductors [11], cigarette additives [9], and predominantly diesel fuel [12]. What is more, CeO_2_-NPs have unique properties that make them good candidates for various medical applications, like drug delivery systems [13], biosensors [14], and imaging techniques [15].

However, naked nanoceria are poorly water-soluble, which restricts its applications for biomedical purposes. Recent investigations suggest the polymer coating of nanoceria as an influential solution to the issues of weak stability, poor biocompatibility, and low water solubility [16]. Poly(allylamine) (PAA) is a cationic polymer with a large number of amino groups and displays great water-soluble and biodegradable properties [17,18]. Several studies support the use of PAA in nanoparticulate systems to enhance their efficiency in designing new therapeutic products [19,20,21]. In this work, based on the positive contribution of PAA to the development of functional materials, a simple sol–gel route was utilized to prepare biocompatible CeO_2_–NPs with anticancer effects. To this end, PAA was used as a capping and/or stabilizing agent, which expands during the calcination process, and protects particles from agglomeration.

## 2. Materials and Methods

### 2.1. Chemicals and Reagents

All the materials used in the present work were of an analytical grade. Cerium nitrate hexahydrate [Ce(NO_3_)·6H_2_O, 99%] was purchased from Samchun (Seoul, Korea), and PAA with three different molecular weights of 65,000 (20 wt.% in H_2_O), 17,000 (20 wt.% in H_2_O), and 15,000 (10 wt.% in H_2_O) g/mol was procured from Polysciences. Ammonium hydroxide (NH_4_OH, 25 vol%; Merck, Darmstadt, Germany), and glacial acetic acid (Merck, Darmstadt, Germany) were used for the synthesis of nanoparticles. HeLa (human cervical adenocarcinoma) and MCF-7 (human breast cancer) cell lines were obtained from the Pasteur Institute of Iran (IPI). Phosphate Buffered Saline (PBS), streptomycin, trypsin, penicillin, dimethyl sulfoxide (DMSO), and 3-(4, 5-Dimethyl-2-thiazolyl)-2, 5-diphenyl-2H-tetrazolium bromide (MTT) were acquired from Sigma-Aldrich (Saint Louis, MO, USA). Fetal Bovine Serum (FBS; 10%) and Dulbecco’s Modified Eagle’s Medium (DMEM) were obtained from GIBCO (Carlsbad, CA, USA). *N*-(2-Hydroxyethyl) piperazine-N’-(2-ethane sulfonic acid) (HEPES) (Sigma-Aldrich, Steinheim, Germany), Triton X-100 (Merck, Darmstadt, Germany), and V-bottom 96-well plate (Sigma-Aldrich, Steinheim, Germany) were utilized to evaluate the hemolytic activity of the CeO_2_-NPs. Double distilled water was used for all experiments.

### 2.2. Synthesis of CeO_2_-NPs

The CeO_2_-NPs were prepared by the sol–gel method using PAA (15,000, 17,000, and 65,000 g/mol) as an organic template. Briefly, 20.0 g of cerium nitrate hexahydrate and 5.0 gr of PAA were added separately to 100 mL of distilled water. Then, the cerium nitrate solution was mixed slowly with the PAA solution by vigorous stirring for 30 min. Afterward, 1 M ammonium hydroxide was added to the solution in a drop-wise fashion until the final pH became approximately 10. Glacial acetic acid was applied to adjust pH in the final solution. The obtained suspension was stirred at 70 °C for 10 h until the citrine color resin (gel-like material) was achieved. The precipitate was finally obtained by centrifuging and washing in order to remove the remaining nitrate, ammonia, and organic impurities. The result of thermogravimetric/differential thermal analysis (TGA/DTA, TA Instruments SDT Q600) revealed that the calcination temperature must be 400 °C. Therefore, the collected samples were heated in an electric muffle furnace (Finetech, Seoul, Korea) at 400 °C at a constant rate of 5 °C/min and maintained at this temperature for 2 h to obtain “light” citrine CeO_2_-NPs.

### 2.3. Characterization of CeO_2_-NPs

The thermal behavior of the synthesized CeO_2_-NPs was analyzed employing a TGA/DrTGA device (TA Instruments SDT Q600). Around 5 mg of the NP samples were heated up to 1000 °C in an air atmosphere at the heating rate of 10 °C/min. Moreover, the NPs were characterized by powder X-ray diffraction (PXRD), Field Emission Scanning Electron Microscope (FESEM), Fourier-transform infrared (FTIR) spectroscopy, and UV-Visible (UV-vis) spectrophotometry. The PXRD was carried out by using a Philips X’pert diffractometer (USA) equipped with Cu Kα (λ = 1.54056 A) radiation in the range between 10° and 80° (2θ). The PXRD patterns of the NPs were obtained at a scan speed of 2°/min. The mean particle size (D) of the prepared CeO_2_-NPs was calculated from the XRD data using the Scherrer equation:

D = 0.94λ/βcosθ(1)

where 0.94 is taken as the shape factor, λ is the X-ray wavelength for CuKα radiation (0.15406 nm), θ is the Bragg diffraction angle, and β is the full-width at half-maximum (FWHM) of the (1 1 1) diffraction peak.

The shape and morphology, along with the particle size distribution of the NPs were recorded by a Carl Zeiss SUPRA 55VP FESEM (Wetzlar, Germany) with an operating voltage in the range of 20–30 kV. The FTIR spectroscopy was conducted within the range of 400–4000 cm^−1^ to determine the surface and chemical composition of the NPs. The spectra were recorded applying an ST-IR\ST-SIR spectrometer (Thermo Scientific, Waltham, MA, USA) after 64 scans. The optical absorption spectra of the NPs were analyzed employing a UV-vis spectrophotometer (Evolution 300^®^ Thermo-Fisher Scientific, Dreieich, Germany) over the range of 200–800 nm.

### 2.4. Evaluation of Cytotoxicity Effects

The cytotoxicity and half-maximal inhibitory concentration (IC50) of the NPs were evaluated by the MTT assay [22]. Briefly, HeLa and MCF7 cell lines were cultured in high glucose DMEM medium (1 g/L glucose, 2 mM glutamine) supplemented by 10% (*v*/*v*) FBS, 100 units/mL penicillin, and 100 μg/mL streptomycin. These cells were then incubated at 37 °C under damp air conditions (90% relative humidity) with 5% CO_2_. After three days, the non-adherent cells were segregated by replacing the medium with a fresh one, while the anchorage-dependent cancer cells were expanded further through two passages. Subsequently, the cells were harvested from culture flasks via treatment with trypsin (0.25%) and seeded overnight at a density of 1 × 10^4^ cells per well in a 96-well plate. After that, the cells were exposed to the various concentrations (10 dilutions) of the NPs for 24 h. In the next step, 10 μL of 5 mg/mL MTT in the PBS buffer were added to each well and allowed for 4-h incubation at 37 °C. Following DMSO (100 μL) addition, optical absorbance was recorded at 540 and 630 nm (reference wavelength) using a plate reader (Stat FAX 2100, Awareness Technology, San Jose, CA, USA). All experiments were carried out five times [23,24].

### 2.5. Hemolytic Activity of CeO_2_-NPs

Human fresh blood was centrifuged at 3000 rpm for 10 min at 4 °C. The cell pellet was rinsed with PBS four times (followed by centrifugation under the mentioned conditions) and then diluted ten-fold in 150 mM NaCl. Moreover, the calcined CeO_2_-NPs at 400 °C were suspended in a stock solution of 1 mg/mL HEPES-buffered saline (HBS; 150 μL). Thus, the various concentrations of the CeO_2_-NPs were prepared in a V-bottom 96-well plate using triplicates. The negative control was 150 μL of HBS, with 150 μL of 1% Triton X-100 acting as the positive control. After that, the erythrocyte mixture (20 μL) was poured into each well, and the plates were incubated for 45 min at 37 °C under continuous shaking. The supernatant (70 μL) was separated by centrifugation at 3000 rpm for 10 min and used for the next evaluation of hemoglobin release. Measurements were performed at 405 nm on a plate reader [25]. One metric for measuring hemolytic activity is HC50, i.e., the concentration of the NPs that kills 50% of RBCs [26].

### 2.6. Statistical Analysis

All data were displayed as a mean ± standard deviation (SD) and further compared running one-way ANOVA. A *p* < 0.05 was defined as the significance level of the present study. GraphPad Prism (Ver. 6.01) and GraphPad InStat (Ver. 3.10) were used to conduct the statistical analyses.

## 3. Results and Discussion

### 3.1. FTIR Spectroscopy

**Figure 1:**
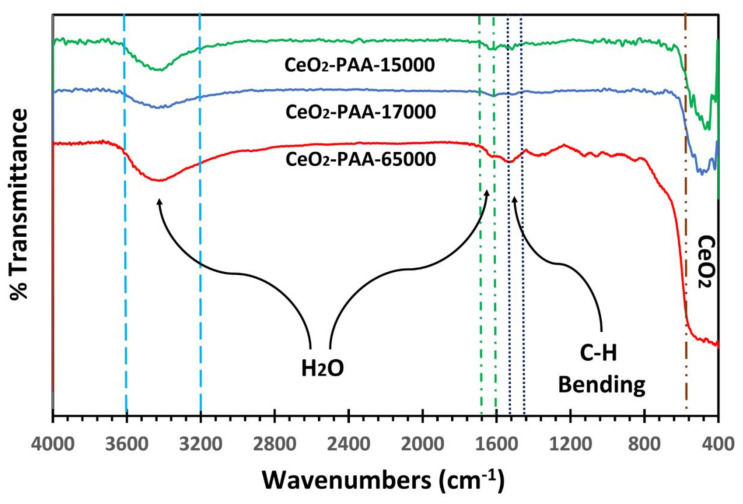
FTIR spectra of CeO_2_–NPs.

The FTIR spectra of the CeO_2_–NPs using PAA15000, PAA17000, and PAA65000 are illustrated in Figure 1. The presence of the cubic CeO_2_ was detected by the FTIR studies. Of the most characteristic bands, there was an intense peak at ~550 cm^−1^ corresponding to the phonon mode of the cubic CeO_2_. The strong absorption peak at 470 cm^−1^ exhibited the Ce-O stretching. In addition, the absorption bands were respectively found at 1620 and 3430 cm^−1^ representing the ν (OH) mode of (H-bonded) water molecules and δ (OH) [27]. No peak for the NO stretch was detected by the FTIR analysis, revealing that the washing procedure omitted the trace amount of nitrate. Furthermore, the absorption peaks in the range of 1400–1500 cm^−1^ corresponded to the bending vibrations of -CH groups [28].

### 3.2. Thermal Analysis

**Figure 2:**
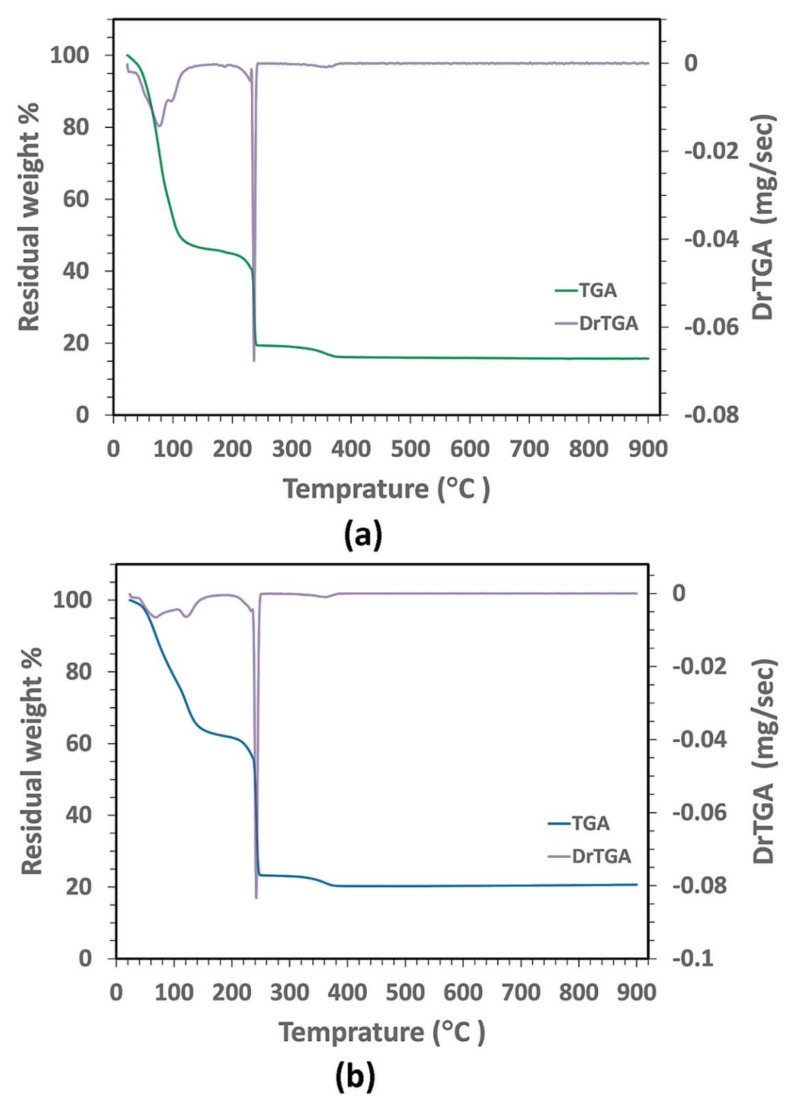
TGA/DrTGA curves of the as-prepared gels with (**a**) PAA15000 and (**b**) PAA17000.

The color of the sol–gel-derived CeO_2_–NPs was changed from brown to white because of the increased calcination temperature. Figure 2 displays the TGA/DrTGA curves of the as-prepared PAA-based gels. Since the same thermal behavior was observed for all molecular weights of PAA, TGA/DrTGA measurements are described for the samples with the PAA molecular weight of 15,000 g/mol. The thermogravimetric analysis showed three main regions of weight loss. The first region, with a weight loss of 55.11% of the initial weight, was in the range between 25 and 185 °C due to the evaporation of the initial water and the partial decomposition of PAA. In the next region, weight loss (26.82% of the initial weight) occurred from 185 to 236 °C as a result of the decomposition of the chemically bonded groups. The final region, from 236 to 462 °C, had a weight loss of 3.4% of the initial weight that may arise from the formation and decomposition of the pyrochlore phases, as well as the crystallization of the CeO_2_ pure phases. There was no weight loss between 462 and 950 °C on the TGA curve, implying that the nanocrystalline CeO_2_ was formed as the decomposition product.

### 3.3. PXRD Analysis

**Figure 3:**
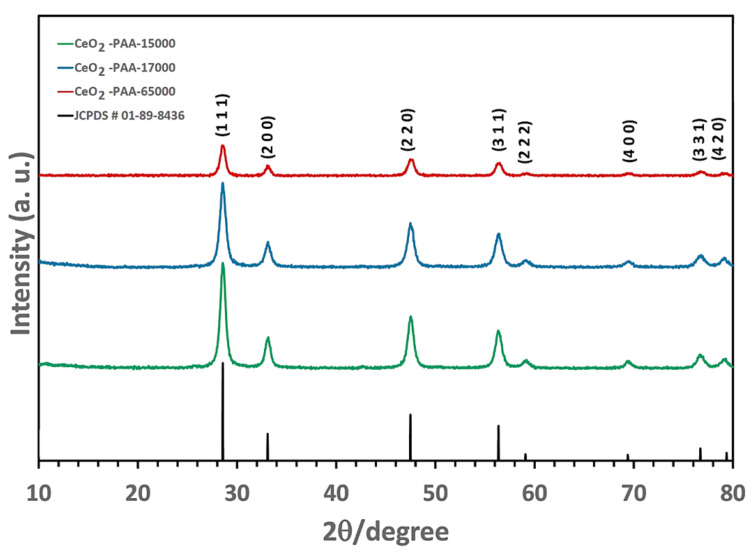
Powder X-ray diffraction (PXRD) patterns of CeO_2_–NPs.

The PXRD patterns of the CeO_2_-NPs are presented in Figure 3. All of the detectable peaks were indexed with Miller indices (hkil = 1 1 1, 2 0 0, 2 2 0, 3 1 1, 2 2 2, 4 0 0, 3 3 1, and 4 2 0), which were consistent with the cubic fluorite-type structure of CeO_2_ (JCPDS # 01-089-8436); this structure had a main orientation of the (1 1 1) reflection plane. The PXRD data confirmed the high quality of the CeO_2_–NPs [no peak indicative of possible intermediate phases, including Ce(OH)_4_/Ce(OH)_3_]. Moreover, it was found that the PXRD peaks became less sharp, and FWHM decreased after calcination at 400 °C for 2 h, suggesting that the crystallinity of the CeO_2_–NPs was deteriorated as the PAA molecular weight increased.

### 3.4. FESEM Analysis

**Figure 4:**
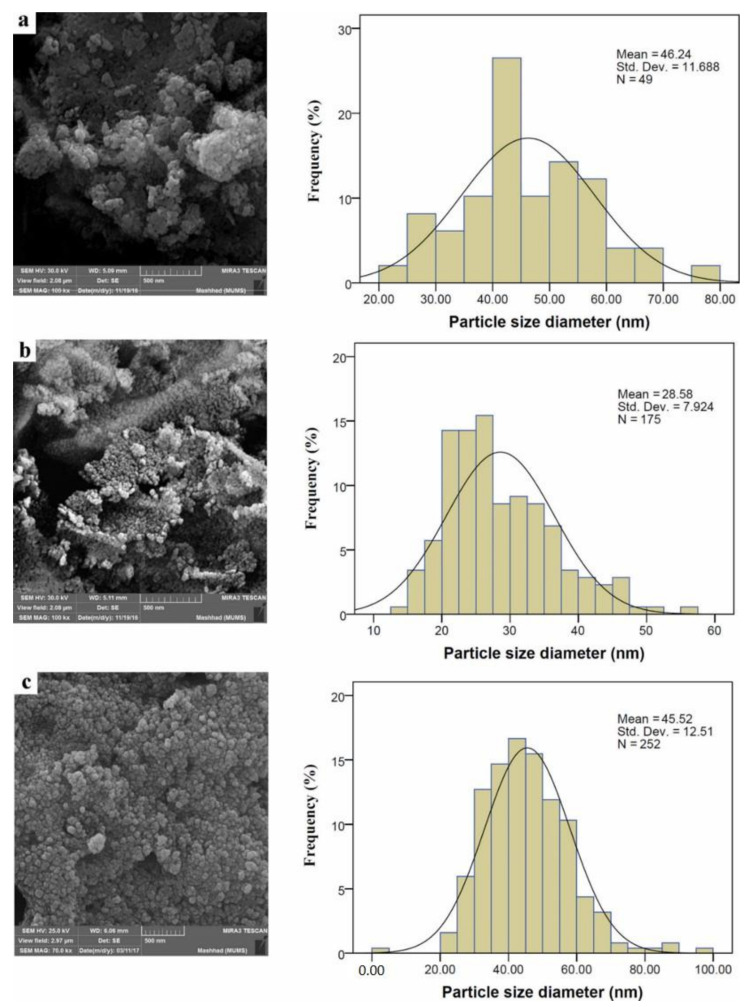
FESEM images of CeO_2_–NP prepared by (**a**) PAA15000, (**b**) PAA17000, (**c**) PAA65000.

The FESEM image and size distribution of the CeO_2_–NPs are demonstrated in Figure 4 at various magnifications. As can be seen, the synthesized CeO_2_–NPs had the form of spherical aggregations. These particles were small in size, with an average size of 46.24, 28.58, and 45.52 nm (less than 50 nm [22]) when PAA15000, PAA17000, and PAA65000 were used, respectively. These findings were in line with the PXRD data and could explain the broadening of the peaks. According to the FESEM images, the nano-sized particles were uniform in morphology, and the use of PAA-65000 led to the NPs with a narrower size distribution.

### 3.5. Optical Properties of Calcined Nanoceria

**Figure 5:**
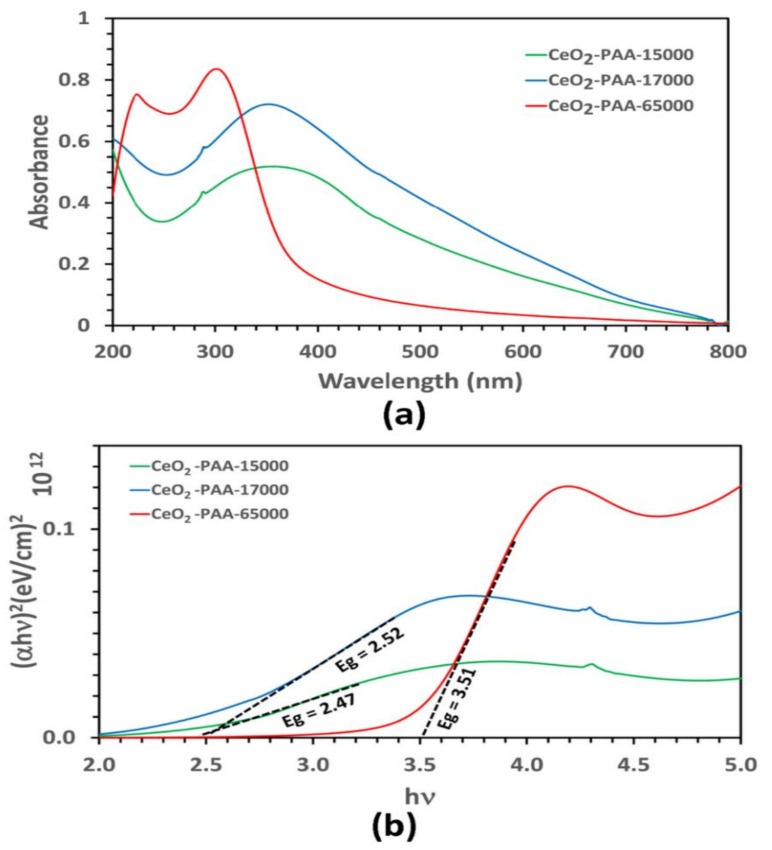
Optical absorption (**a**) and Tauc plot (**b**) of CeO_2_-NPs.

In Figure 5, the UV-vis spectra exhibited a strong absorption peak at <299 nm for the CeO_2_–NPs aqueous dispersion with PAA65000, which is evidence for the presence of Ce^3+^ on the surface of the nanoceria. On the contrary, the CeO_2_–NPs aqueous dispersion with PAA15000 and PAA17000 had a characteristic absorption peak at >360. In the pertinent literature, it has been reported that Ce^3+^ and Ce^4+^ absorb light within the range of 230–260 nm and 300–400 nm, respectively [29]. On the whole, the oxidized state of cerium (Ce^4+^) constitutes the main content of a nanoceria crystal. When the crystal size reduces, the surface defects linked to oxygen vacancies elevate, which, in turn, raises Ce^3+^ on the surface. The ratio of Ce^4+^/Ce^3+^ plays a pivotal role in the mechanism whereby nanoceria scavenge reactive oxygen species (ROS) [30]. The absorption peak in the UV region is more likely to arise from the electronic transitions corresponding to the electronic excitation of O(2p) state to Ce(4f) orbitals and the spin–orbit coupling of 3d orbitals [22,31]. What is more, the sharp peak for the CeO_2_–NPs prepared by PA65000 confirmed their narrow size distribution. Tauc’s equation was used to estimate the band gap energy of calcined CeO_2_–NPs:

(αhν)^n^ = B(hν-E_g_) (2)

where B is a constant, hν is the photon energy, α is the absorption coefficient, n is 2 for the direct transitions, and E_g_ (i.e., band gap energy) is the spot of the junction of the Tau plot slope on the x-axis [32,33]. According to Figure 5b, the calculated band gap energies for the CeO_2_-NPs prepared by PAA15000 and PAA17000 were 2.47 and 2.52 eV, respectively. These samples showed a strong red shift in the band gap as compared with the bulk cerium oxide (E_g_ = 3.19 eV) [34]. On the other hand, the samples with PAA65000 had a band gap energy of 3.51 eV, greater than that of the bulk CeO_2_, and indicative of the blue shift in the band gap as a result of quantum confinement [35]. A decrease in the PAA molecular weight from 65,000 g/mol to 15,000 g/mol was obviously associated with a red shift in λ_max_, which could be due to agglomeration and wide particle size distribution. This observation can be explained partly by the fact that typical direct semiconductors indicate a blue shift in λ_max_ if their particle sizes reduce [33,36]. λ_max_ and crystallite size are summarized in Table 1.

### 3.6. Biocompatibility of the Synthesized Samples

**Figure 6:**
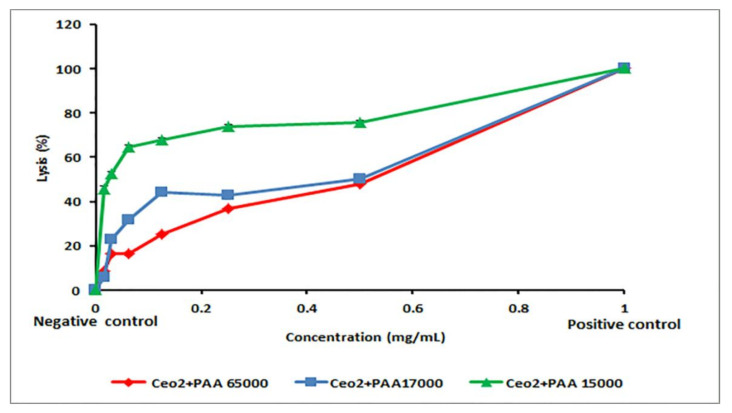
Hemolysis activity of CeO_2_-NPs.

In the human body, the erythrocytes exist in great quantities and possess large amounts of biological and morphological characteristics. Therefore, these cells have been vastly used in drug transport. The main target of the polyunsaturated fatty acids as well as hemoglobin molecules, known as redox-active oxygen transport molecules and potent promoters of activated oxygen species, is the erythrocytes. Oxidative damage to their membrane lipids and proteins can account for hemolysis that further causes several complications, such as oxidative drugs and excess of transition metals [37]. Moreover, hemolysis is more likely to induce anemia in different medical conditions [38,39]. This experiment was applied to evaluate if synthesized nanoceria could mutilate the erythrocyte membrane, given some previous reports concerning the toxicity of the CeO_2_-NPs [40,41]. The results in Figure 6 indicated that the CeO_2_–NPs prepared by PAA had potent hemolytic actions in a dose-dependent manner. The lysis of erythrocytes was found to decrease significantly as the concentration of the NPs increased (*p* < 0.001). At an identical concentration, the CeO_2_–NPs with PAA15000 exhibited maximum hemolysis, whereas the CeO_2_–NPs with PAA65000 inhibited hemolytic activity to the greatest extent. The HC50 values are shown in Table 2. The highest HC50 was for the sample prepared with PAA65000 (7.35 ± 1.32 mg/mL). This finding implies that less damage occurred following the use of the CeO_2_-NPs with PAA65000, which may be attributed to their λ_max_ < 299 nm and high amounts of Ce^3+^ on the surface. Besides, this in vitro test revealed the biocompatibility of the synthesis of nanoceria using PAA65000, which makes them promising for future drug development and biomedical applications, such as the treatment of cancer.

### 3.7. Anticancer Activity of Nanoceria

**Figure 7:**
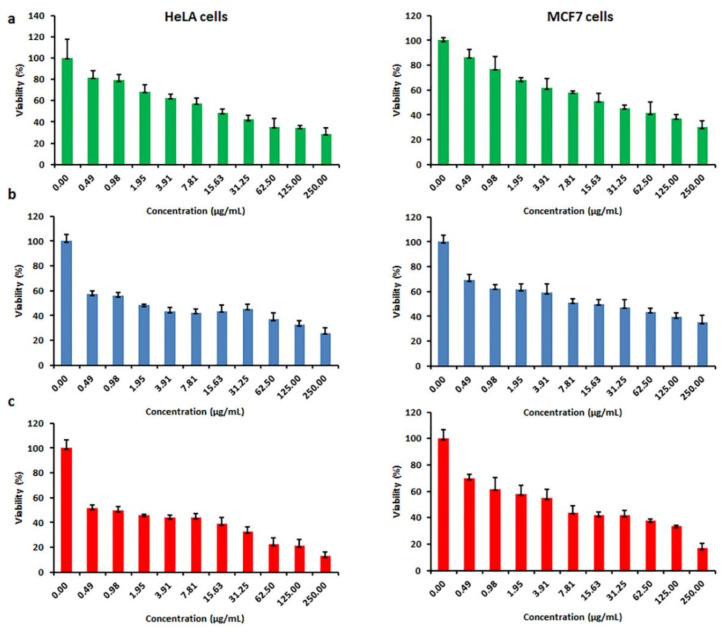
In vitro cytotoxicity of CeO_2_–NPs after 24 h of incubation with different concentrations of CeO_2_–NP prepared by (**a**) PAA15000, (**b**) PAA17000, (**c**) PAA65000.

A body of literature shows nanoceria acts as a regenerative radical scavenger that can produce the active Ce^3+^ oxidation state for inhibition of free radicals. This characteristic differentiates them from other nano-sized materials with antioxidant effects, namely hydroxylated and water-soluble C-60 and SWCNTs [42,43,44]. Free radicals and ROS are found to be involved throughout degenerative processes, like cancer, mostly owing to their capacity to impair cell membranes, DNA, and proteins [45,46]. Hence, nanoceria can afford to block the production of ROS and free radicals and prevent free radical-associated diseases. Nanoceria seem to carry anticancer effects in some cellular models by the mechanisms of oxidative stress and apoptosis while protecting normal cells [34]. In this study, the anticancer properties of the nanoceria against two cancer cell lines, the breast cancer cell line (MCF-7) and cervical carcinoma cell line (HeLa), were evaluated. In Figure 7, the results of cytotoxicity are shown at the concentration between 0.50 and 250 μg/mL of the CeO_2_-NPs. Overall, as the concentrations of the nanoceria increased, the viability of both cancer cells decreased significantly (*p* < 0.001). The proliferation of MCF-7 was affected by 50% at 17.44 ± 7.32, 6.17 ± 1.68, and 0.12 ± 0.03 μg/mL for the samples with PAA15000, PAA17000, and PAA65000, respectively. When HeLa cells were exposed to the nanoceria prepared by PAA15000, PAA17000, and PAA65000, the respective IC50 values were 8.09 ± 1.55, 2.11 ± 0.33, and 0.20 ± 0.01 μg/mL. Therefore, not only could the CeO_2_-NPs with PAA65000 counteract the growth of cancerous cells effectively, but almost no lysis also occurred in RBCs at 0.12 ± 0.03 and 0.20 ± 0.01 μg/mL.

## 4. Conclusions

The CeO_2_-NPs were prepared by a sol–gel method, using PAA with different molecular weights (15,000, 17,000, and 65,000 g/mL) as an organic template. The structural analyses exhibited that the calcined ceria at 400 °C had nano-sized particles <50 nm. The FTIR spectroscopy confirmed no trace amount of nitrate that can explain the notable biocompatibility of the NPs. Moreover, the CeO_2_-NPs had anticancer effects on the viability of both MCF7 and HeLa cells. The NPs prepared by the use of PAA65000 resulted in a narrower distribution of particle size, marked cytotoxicity against cancer cell lines, and almost no RBC lysis at IC50. The in vitro test on erythrocytes revealed the biocompatibility of the CeO_2_-NPs prepared with PAA65000, which makes them a good candidate for prospective biomedical applications, such as cancer therapy.

## Figures and Tables

**Table 1:** Molecular weight, optical properties, and particle size of nanoceria.

PAA Molecular Weight in CeO_2_-NPs, g/mole	λ_max_, nm	E_g_, eV	Crystallite Size (nm)	
			Calculated ^1^	Observed ^2^
15,000	348	2.47	12.26	46.24
17,000	354	2.52	13.66	28.58
65,000	298	3.51	15.04	45.52

^1^ The mean crystallite sizes (D) of CeO_2_–NPs were determined by the Debye–Scherrer formula. ^2^ The mean particle sizes of CeO_2_–NPs were obtained by the FESEM images.

**Table 2:** Molecular weight, 50% hemolytic concentration (HC50), and half-maximal inhibitory concentration (IC50) of nanoceria.

PAA Molecular Weight in CeO_2_-NPs, g/mole	HC50 (mg/mL)	IC50 (μg/mL)	
		MCF-7	HeLa
15,000	0.022 ± 0.001	17.44 ± 7.32	8.09 ± 1.55
17,000	3.74 ± 0.58	6.17 ± 1.01	2.11 ± 0.33
65,000	7.35 ± 1.32	0.12 ± 0.03	0.20 ± 0.01
